# Type-5 diabetes mellitus: a call for clinical recognition and research prioritization

**DOI:** 10.1097/MS9.0000000000004912

**Published:** 2026-04-02

**Authors:** Zain Ul Abedeen, Ayesha Saleem, Abad Ur Rehman

**Affiliations:** a Department of Internal Medicine, Saidu Medical College, Swat, Pakistan; b Department of Medicine, Spinghar University (Kabul campus), Kabul, Afghanistan

*Dear Editor*,

Diabetes mellitus comprises a group of disorders characterized by disrupted carbohydrate metabolism, resulting in chronic hyperglycemia in the presence of impaired glucose utilization. According to the American Diabetes Association, diabetes is classified into several clinical categories: type 1 involves the immune-mediated destruction of pancreatic beta cells, type 2 is characterized by insulin resistance, gestational diabetes occurs during pregnancy, and the secondary types arise due to other causes^[^1^]^.

A historically neglected, misdiagnosed, and poorly understood type, type 5 diabetes mellitus (T5DM) was formally recognized during the 2025 World Diabetes Congress of the International Diabetes Federation (IDF)^[^2^]^. Patients with T5DM typically lack insulin resistance and markers of autoimmunity, have decreased insulin secretion resulting in persistent hyperglycemia, yet they do not develop ketosis and ketonuria, which makes it distinct from the other types. It is believed to originate from impaired pancreatic tissue development due to chronic malnutrition, and has therefore also been referred to as malnutrition-related diabetes mellitus (MRDM)^[^3,4^]^. Experimental studies on rodents and primates have shown that malnourishment during critical periods of growth – prenatal and early life – results in impaired pancreatic β-cell proliferation, dysfunctional mitochondria, and influences epigenetics of pancreatic islets. Overall, the outcome is reduced β-cell mass and decreased insulin secretory capacity. These mechanistic findings support the hypothesis that T5DM, characterized by a lifelong state of insulin deficiency, stems from chronic malnutrition^[^5,6^]^. Clinically, it has a type 1-like presentation and has, thus, been reported to be misdiagnosed and mistreated as type 1 diabetes^[^2,7^]^.

Recent syntheses and population studies estimate that T5DM effects on the order of 20–25 million individuals worldwide, predominantly affecting lean, young populations in low- and middle-income regions such as South-East Asia and sub-Saharan Africa^[^3,8^]^. Despite its high prevalence, it remains underreported and has been rarely discussed. It could be attributed to the fact that the majority of research data and medical literature come from the West and developed countries, where malnutrition is rare, and hence, this type of diabetes is not prevalent. Many studies in the past have reported atypical presentations of diabetes in middle- and low-income countries^[^4,7,9^]^. It was first reported in 1955 in Jamaica and termed “type J diabetes.” Subsequently, different terms have been used to describe T5DM-like presentations, including tropical pancreatic diabetes (TPD), protein-deficient pancreatic diabetes (PDPD), and phasic insulin-dependent diabetes, among others^[^7^]^. The World Health Organization (WHO) admitted the role of malnutrition in diabetes in 1965 and officially recognized MRDM as a distinct type in 1985, but they later removed it from classifications due to scarce evidence^[^2,7,9^]^. It has been overlooked for the past 70 years due to a lack of evidence and unreported epidemiological data. It has always been there, affecting millions, yet it has remained unrecognized.

The IDF’s recognition of T5DM marks a decisive step toward addressing past negligence. It highlights the lack of research in developing countries and calls for a reassessment of global research priorities. Academic institutions, funding bodies, and journals should prioritize studies related to this entity, which will aid in elaborating on it, as well as in establishing diagnostic criteria and therapeutic guidelines. Clinicians should identify and report such atypical presentations of common disorders. There may be many more similarly overlooked diseases yet to be recognized. As a pragmatic starting point for clinical recognition and future guideline development, we propose that clinicians consider the following preliminary features when evaluating patients for T5DM: (1) onset in adolescence or young adulthood with a lean phenotype and a history consistent with chronic undernutrition; (2) absence of pancreatic autoantibodies (e.g., GAD65, IA-2); (3) evidence of insulin deficiency (low fasting/stimulated C-peptide) in the presence of preserved insulin sensitivity; and (4) absence of ketosis/ketonuria despite hyperglycemia. For management, initial emphasis should be on nutritional rehabilitation, careful glycemic control with cautious insulin dosing where necessary, and prospective evaluation of oral agents in research settings. These proposals are provisional and intended to guide research and case ascertainment rather than to replace formal diagnostic consensus developed through multicenter studies.

## Data Availability

All the data used in the study are available online and can be accessed through the reference list.
